# Identification of Novel Biomarkers in Pancreatic Tumor Tissue to Predict Response to Neoadjuvant Chemotherapy

**DOI:** 10.3389/fonc.2020.00237

**Published:** 2020-03-04

**Authors:** Sumit Sahni, Christopher Nahm, Christoph Krisp, Mark P. Molloy, Shreya Mehta, Sarah Maloney, Malinda Itchins, Nick Pavlakis, Stephen Clarke, David Chan, Anthony J. Gill, Viive M. Howell, Jaswinder Samra, Anubhav Mittal

**Affiliations:** ^1^Northern Clinical School, Faculty of Medicine and Health, University of Sydney, Camperdown, NSW, Australia; ^2^Bill Walsh Translational Cancer Research Laboratory, Kolling Institute of Medical Research, University of Sydney, Camperdown, NSW, Australia; ^3^Australian Pancreatic Centre, Sydney, NSW, Australia; ^4^Center for Diagnostics, Clinical Chemistry and Laboratory Medicine, University Medical Center Hamburg – Eppendorf, Hamburg, Germany; ^5^Bowel Cancer and Biomarker Research Laboratory, Kolling Institute of Medical Research, Royal North Shore Hospital, St Leonards, NSW, Australia; ^6^Australian Proteome Analysis Facility (APAF), Macquarie University, Sydney, NSW, Australia; ^7^Northern Sydney Cancer Center, Royal North Shore Hospital, St Leonards, NSW, Australia; ^8^Northern Cancer Institute, St Leonards and Frenchs Forest, St Leonards, NSW, Australia; ^9^Cancer Diagnosis and Pathology Group, Kolling Institute of Medical Research, Royal North Shore Hospital, St Leonards, NSW, Australia; ^10^Upper GI Surgical Unit, Royal North Shore Hospital and North Shore Private Hospital, Sydney, NSW, Australia

**Keywords:** pancreatic ductal adenocarcinoma, biomarkers, neoadjuvant chemotherapy, proteomics, SWATH-MS

## Abstract

**Background:** Neoadjuvant chemotherapy (NAC) has been of recent interest as an alternative to upfront surgery followed by adjuvant chemotherapy in patients with pancreatic ductal adenocarcinoma (PDAC). However, a subset of patients does not respond to NAC and may have been better managed by upfront surgery. Hence, there is an unmet need for accurate biomarkers for predicting NAC response in PDAC. We aimed to identify upregulated proteins in tumor tissue from poor- and good-NAC responders.

**Methods:** Tumor and adjacent pancreas tissue samples were obtained following surgical resection from NAC-treated PDAC patients. SWATH-MS proteomic analysis was performed to identify and quantify proteins in tissue samples. Statistical analysis was performed to identify biomarkers for NAC response. Pathway analysis was performed to characterize affected canonical pathways in good- and poor-NAC responders.

**Results:** A total of 3,156 proteins were identified, with 19 being were significantly upregulated in poor-responders compared to good-responders (log_2_ ratio > 2, *p* < 0.05). Those with the greatest ability to predict poor-NAC response were GRP78, CADM1, PGES2, and RUXF. Notably, canonical pathways that were significantly upregulated in good-responders included acute phase signaling and macrophage activation, indicating a heightened immune response in these patients.

**Conclusion:** A novel biomarker signature for poor-NAC response in PDAC was identified.

## Introduction

Pancreatic ductal adenocarcinoma (PDAC) has the lowest survival rate of all major cancers (~6% at 5 years post-diagnosis) and is projected to become the second most common cause of cancer related death by 2030 (1, 2). Intrinsic chemotherapy-resistance is one of the major clinical problems associated with PDAC, resulting in the failure of currently available therapeutic options (3). Adjuvant chemotherapy in patients with resected PDAC has been shown to extend survival over surgery alone, and more recently, more intensive regimens such as FOLFIRONOX have been shown to be even more effective (4). However, not all patients are capable of commencing let alone completing chemotherapy after surgery for PDAC. As such, there has been an increasing trend toward neoadjuvant chemotherapy (NAC), i.e., pre-operative chemotherapy, in order to effectively deliver systemic chemotherapy since improvements in nodal status and resection margin status have been observed (5, 6). However, a subset of patients can be classified as “poor responders” to NAC, failing to demonstrate tumor response with subsequent early disease recurrence and shortened overall survival time (7). While genetic classification of PDAC may help identify a high risk squamous or basal subtype (8), the high costs of these methodologies have prohibited the general clinical use of genetic analysis of individual PDAC patients to help guide therapy. Therefore, there is a need for discovering more readily applicable tissue and/or blood-based secreted biomarkers that can predict a NAC response, which may be detected by more cost-effective tests.

Recently, there has been a surge in interest in the “-omics” approach to biomarker discovery in cancer research. Such approaches allow identification of a myriad of genes, transcripts, proteins and metabolites unique to cancer. They are therefore an invaluable first-step in the process of biomarker identification and validation. SWATH-MS (Sequential Window Acquisition of all Theoretical fragment ion spectra—Mass Spectroscopy) is a high throughput quantitative mass spectrometry method for proteome analysis (9). This technology allows permanent recording of all peptide fragment ions in biological samples, which imparts the advantages of a high throughput shotgun approach, with the consistency and data reproducibility of selective reaction monitoring (SRM) proteomics (10). Here, we report on the use of SWATH-MS as a discovery proteomics approach to identify differences in proteomic profile of good- and poor-NAC responders in PDAC.

## Materials and Methods

### Participants and Tissue Collection

Patients who presented with histologically confirmed PDAC at a tertiary centre [Royal North Shore Hospital (RNSH) and North Shore Private Hospital (NSP), Sydney, Australia] were included in the study between 04/03/2016 and 18/07/2017. All patients selected were treated with NAC before surgical resection, following individual discussion by our multidisciplinary team. The NAC regimen was at the discretion of the oncologist. Tumor tissue and adjacent normal pancreas were obtained from patients during the surgical intervention. Pathologically confirmed tumor and adjacent normal pancreas tissue were cut into 2 mm^3^ portions and stored in cryotubes in a −80°C freezer for later analysis.

The NAC response was determined based on the residual tumor viability, as described previously (7). Briefly, at the time of initial surgical pathology reporting, the residual tumor viability was assessed by the reporting pathologist. All histological slides were reviewed to estimate the viable residual tumor as a percentage of the estimated original tumor volume. A case with no response to NAC was recorded as 100% viable and a case with complete regression after treatment was recorded 0% viable.

### Ethics Approval and Consent to Participate

This study was approved by the RNSH and NSP institutional ethics committees under references HREC/16/HAWKE/105 and NSPHEC 2016-007, respectively. Informed written consent was obtained from all participants and/or their designated surrogate. North Sydney Local Health District (NSLHD) reference: RESP/16/76.

### Proteomic Sample Preparation and SWATH-MS Analysis

#### Protein Digestion and LC-MS/MS Analysis

All tissue samples were lysed in 100 mM triethylammonium bicarbonate (TEAB) and 1% sodium deoxycholate buffer using a probe sonicator. Protein concentrations were estimated using the bicinchoninic acid protein assay (Thermo Scientific, Waltham, MA). The cysteine residues were reduced in the presence of 10 mM dithiothreitol (DTT, Bio-Rad, Hercules, CA) at 60°C and alkylated with 10 mM iodoacetamide (IAA, Bio-Rad) at room temperature in the dark. Trypsin (sequencing grade; Promega, Madison, WI) was added in a 1:50 ratio and proteins were enzymatically degraded overnight at 37°C. By adding 1 μL formic acid (FA; Thermo Scientific) the digestion was quenched and the sodium deoxycholate (SDC) precipitated and removed by centrifugation (14,000 rpm) for 5 min. Samples were lyophilized and reconstituted in 2% acetonitrile (ACN; Sigma Aldrich, St. Louise, MO) and 0.1% FA.

Liquid Chromatography-Tandem Mass Spectrometry (LC-MS/MS) analysis for tissue samples were performed on an Ekspert NanoLC 400 with cHiPLC system (SCIEX, Framingham, MA) coupled to a TripleTOF 6600 mass spectrometer (SCIEX). A 200 μm × 0.5 mm nano cHiPLC trap column and 15 cm × 200 μm nano cHiPLC columns (ChromXP™ C18-CL 3 μm 120 Å) were used with 140 min ACN gradients.

Digested samples were pooled, by combining a small fraction of each tissue sample from the tumor and adjacent normal pancreas, and subjected them to basic reverse phase chromatography high performance liquid chromatography (HPLC), using an extended C18 column 2.1 mm × 150 mm, 3.5 μm (Agilent, Santa Clara, CA), on an Agilent 1200 series HPLC. One hundred microgram of peptides per pool were pre-cleaned with Sep-Pak C18 and then injected at a flow rate of 0.3 mL/min at room temperature onto the column. The peptides were separated over a 1 h gradient from using Buffer A of 5 mM ammonia at approximately pH 10.4 and Buffer B of 90% ACN/5 mM ammonia, and eluting peptides were collected in fractions of 1 min. Concatenated pooling of the fractions was performed.

For data dependent MS/MS acquisition to build a spectral library of the basic reverse phase fractionated samples, the 20 most intense m/z values exceeding a threshold >250 cps on the TripleTOF 6600 with charge stages between 2+ and 4+ were selected for analysis from a full MS survey scan and excluded from analysis for 20 s to minimize redundant precursor sampling.

In data independent acquisition, a 100 variable window method was used over a range of 400–1,250 m/z with window sizes based on precursor densities in the LC-MS/MS acquisition. Collision energies were calculated for 2+ precursors with m/z values of lowest m/z in window + 10% of the window width. The data were acquired over an 80 min ACN gradient.

#### Protein Identification and Quantification

Spectral libraries for SWATH-MS quantitation were generated with ProteinPilot™ software 5.0 using the Paragon™ algorithm (SCIEX) in the thorough ID mode including biological modifications and chemical modifications. MS/MS data were searched against the human UniProt database (release February 2016, 20,198 entries) with carbamidomethyl as a fixed modification for cysteine residues. An Unused Score cut-off was set to 0.05 and the false discovery rate (FDR) analysis was enabled.

Generated Paragon group files were imported into PeakView™ software 2.1 using the SWATH MicroApp 2.0 (release 25/08/2014) to generate a sample specific spectral library which was matched against SWATH-MS data. After retention time calibration with endogenous peptides, data were processed using following processing settings; 100 maximal peptides per protein, maximal 6 transitions per peptide, peptide confidence threshold of 99%, transition false discovery rate < 1%, 5 min extraction window and fragment extraction tolerance of 75 ppm.

### Data Analysis

Survival data was compared using Kaplan-Meier curve analysis. The statistical differences in the survival curve were analyzed by the Log-rank test. Proteomic data was initially analyzed by the principal component analysis (PCA) to observe inherent groupings within the data set. Further, proteins which were markedly up- or down-regulated (log_2_ ≥ 2 or ≤ −2) were compared using multiple *t*-test analysis (*p* < 0.05; *q* < 0.1; false discovery rate was determined with *Q* = 1%). The predictive model for selected proteins was validated by the Area Under the Receiver Operating Characteristic (AUROC) curve. All analysis was performed using either GraphPad Prism (GraphPad Software, San Diego, California) or JMP (SAS Institute, Cary, North Carolina) statistical software. Pathway analysis was performed using Ingenuity Pathway Analysis (IPA; Qiagen Bioinformatics, Redwood City, CA) (11). The proteins which were markedly (log_2_ ≥ 2 or ≤ −2) and significantly (*p* < 0.05; *q* < 0.1) differentially expressed were inputted into IPA. Protein secretion prediction was performed using Proteinside software (12).

## Results

### Population Demographics and Survival Data

**Figure 1:**
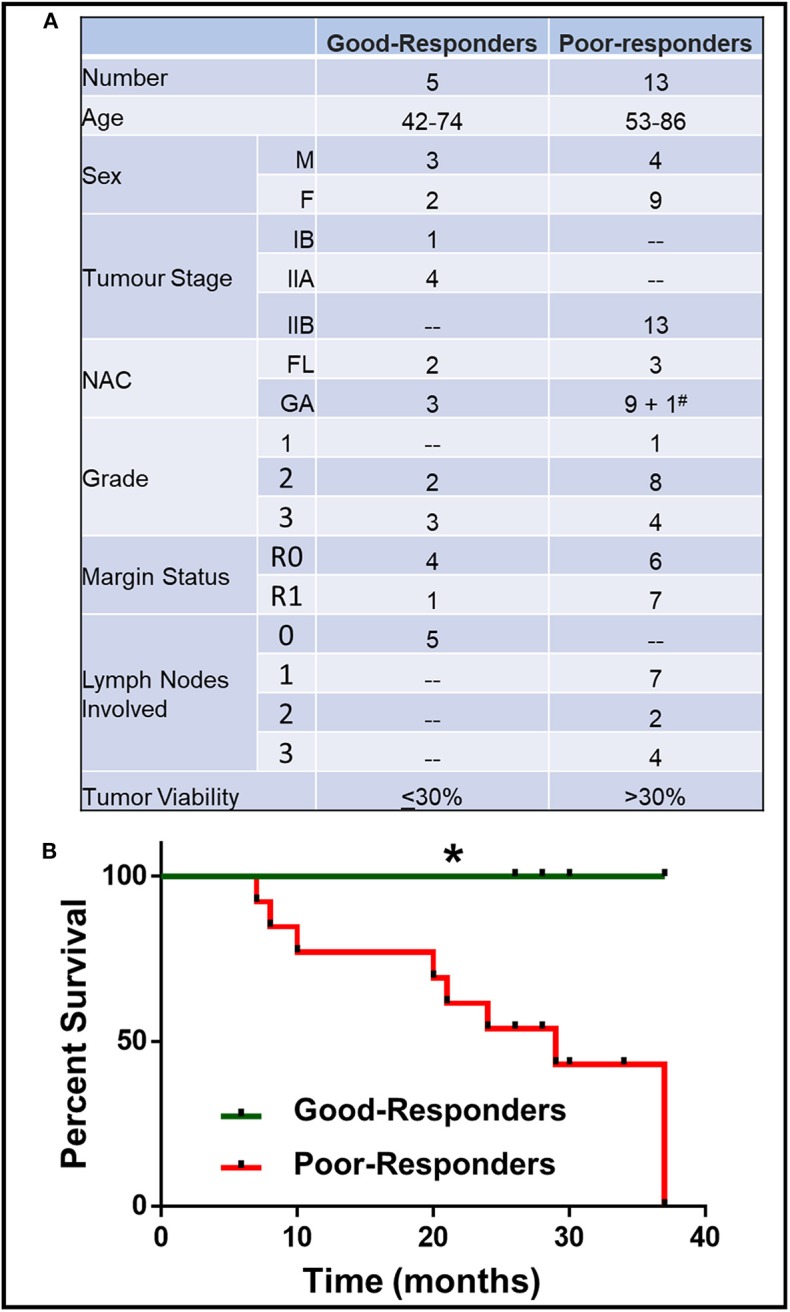
Characteristics of patient with good and poor NAC response. **(A)** Details of patient age, sex, tumor stage, grade, margin status, number of lymph nodes involved, neoadjuvant chemotherapy (NAC) received (FL, Florfirinox; GA, Gemcitabine/Abraxane; ^#^Patient initially received FL followed by GA) and residual tumor cell viability. **(B)** Kaplan-Meier survival curve for good- and poor-NAC responders. *p < 0.05.

A total of 18 PDAC patients (7 males, 11 females) were recruited for this study. All PDAC patients underwent neoadjuvant chemotherapy (NAC) before surgical resection. Patient characteristics (age, sex, tumor stage, NAC received, residual tumor viability) are described in Figure 1A.

The patients were divided on the basis of their response to NAC, which was determined by the residual tumor viability in the specimen. Based on the previously described classification methods (13), the tumors with ≤30% viable tumor cells (i.e., HTRG grade 0, CAP grade 0; HTRG grade 1, CAP grade 1; and HTRG grade 2, CAP grade 2: complete to moderate response) were graded “good-responders,” while tumors with >30% viable tumor cells (HTRG grade 2, CAP grade 3; poor response) were graded as “poor-responders.” The good-responders had significantly (*p* < 0.05) longer overall survival compared to poor-responders (Figure 1B).

### Principal Component Analysis: Distinct Tissue Samples

**Figure 2:**
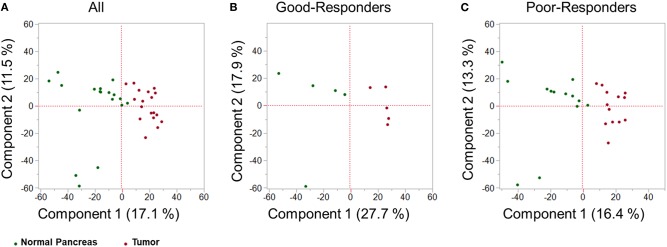
Multivariate proteomic analysis. Principal Component Analysis (PCA) score plot between first two principal components derived from the proteomic profile of tumor tissue (red) and adjacent healthy pancreas (green) in: **(A)** all PDAC patients; **(B)** good-NAC responders; and **(C)** poor-NAC responders.

Using SWATH-MS analysis, a total of 3,156 proteins were identified in both tumor tissue and adjacent normal pancreas. Principal component analysis (PCA) was performed on the proteomic data obtained by SWATH-MS analysis of tumor tissue and adjacent normal pancreas. PCA is an unsupervised class recognition approach, to observe inherent groupings (14). Tissues were observed to be clustered according to their class grouping (i.e., tumor tissue or adjacent normal pancreas) for all patients together (Figure 2A), good-responders (Figure 2B), or poor-responders (Figure 2C). These results indicate that a clearly distinct tumor and adjacent normal tissue specimens were obtained from the patients.

### Differentially Regulated Proteins

There were 236 differentially expressed (log_2_ > 2; *p* < 0.05) proteins in the tumor tissue in good-responders compared to their adjacent normal pancreas (Supplementary Table 1). Of these, 134 proteins were over-expressed and 102 proteins were under-expressed in the tumor tissue. In poor-responders, only 67 proteins were differentially expressed (23 over-expressed and 44 under-expressed; Supplementary Table 2).

The top 10 over- and under-expressed proteins for both good- and poor-responders based on fold-change are reported in Tables 1, 2. The over-expressed proteins in good- and poor-responders showed distinct functional activity. In contrast, the majority of proteins which were under-expressed in both good- and poor-responders, shared similar functional (proteases or peptidase) activity with 7 out of top 10 proteins being the same.

**Table 1:** Over-expressed and under-expressed proteins in good-responders.

**Good-responders**				
**Protein name**	**Uniprot accession**	**Log**_**2**_ **ratio**	***P*****-value**	***q*****-value**
**OVER-EXPRESSED**				
Rho guanine nucleotide exchange factor 18	ARHGI_HUMAN	4.915	0.004	0.011
CDP-diacylglycerol–glycerol-3-phosphate 3-phosphatidyltransferase	PGS1_HUMAN	4.222	0.003	0.010
Prolargin	PRELP_HUMAN	4.115	0.009	0.017
Versican core protein	CSPG2_HUMAN	4.109	0.003	0.010
Alpha-1-antitrypsin	A1AT_HUMAN	3.935	0.007	0.015
Apolipoprotein A-I	APOA1_HUMAN	3.735	0.003	0.010
Hemopexin	HEMO_HUMAN	3.590	0.006	0.014
Collagen alpha-1(III) chain	CO3A1_HUMAN	3.588	0.012	0.019
Inter-alpha-trypsin inhibitor heavy chain H1	ITIH1_HUMAN	3.538	0.005	0.012
Fibulin-1	FBLN1_HUMAN	3.457	0.007	0.015
**UNDER-EXPRESSED**				
Pancreatic alpha-amylase	AMYP_HUMAN	−6.333	4.19E-05	3.14E-03
Chymotrypsin-like elastase family member 3A	CEL3A_HUMAN	−6.245	4.48E-05	3.14E-03
Carboxypeptidase B	CBPB1_HUMAN	−5.428	5.75E-05	3.14E-03
Trypsin-1	TRY1_HUMAN	−5.346	1.26E-03	8.63E-03
Pancreatic lipase-related protein 2	LIPR2_HUMAN	−4.847	3.67E-04	6.36E-03
Bile salt-activated lipase	CEL_HUMAN	−4.775	2.50E-03	9.68E-03
Protein disulfide-isomerase A2	PDIA2_HUMAN	−4.757	2.52E-03	9.68E-03
Carboxypeptidase A1	CBPA1_HUMAN	−4.748	1.18E-02	1.85E-02
Carboxypeptidase A2	CBPA2_HUMAN	−4.395	1.25E-03	8.63E-03
Chymotrypsin-C	CTRC_HUMAN	−4.249	3.48E-03	1.03E-02

**Table 2:** Over-expressed and under-expressed proteins in poor-responders.

**Poor-responders**				
**Protein name**	**Uniprot accession**	**Log**_**2**_ **ratio**	***P*****-value**	***q*****-value**
**OVER-EXPRESSED**				
Periostin	POSTN_HUMAN	2.789	6.94E-06	1.45E-05
Filamin-A	FLNA_HUMAN	2.700	3.44E-05	5.12E-05
Ras-related C3 botulinum toxin substrate 2	RAC2_HUMAN	2.613	2.80E-07	9.38E-07
Proteasome subunit beta type-10	PSB10_HUMAN	2.555	3.31E-05	5.04E-05
Collagen alpha-1(XII) chain	COCA1_HUMAN	2.482	2.64E-07	9.31E-07
Versican core protein	CSPG2_HUMAN	2.436	6.80E-07	1.98E-06
Collagen alpha-2(V) chain	CO5A2_HUMAN	2.388	3.71E-04	4.60E-04
CDP-diacylglycerol–glycerol-3-phosphate 3-phosphatidyltransferase	PGS1_HUMAN	2.373	4.31E-04	5.25E-04
Apolipoprotein A-I	APOA1_HUMAN	2.354	7.56E-04	8.30E-04
Syntenin-1	SDCB1_HUMAN	2.345	6.97E-05	9.16E-05
**UNDER-EXPRESSED**				
Trypsin-3	TRY3_HUMAN	−5.124	1.41E-08	1.58E-07
Chymotrypsinogen B2	CTRB2_HUMAN	−5.067	4.56E-05	6.49E-05
Pancreatic alpha-amylase	AMYP_HUMAN	−4.988	3.63E-08	2.70E-07
Chymotrypsin-like elastase family member 3A	CEL3A_HUMAN	−4.933	2.35E-09	5.57E-08
Protein disulfide-isomerase A2	PDIA2_HUMAN	−4.859	2.19E-08	1.84E-07
Trypsin-1	TRY1_HUMAN	−4.471	2.18E-09	5.57E-08
Carboxypeptidase A1	CBPA1_HUMAN	−4.463	4.03E-06	8.72E-06
Chymotrypsin-C	CTRC_HUMAN	−4.390	2.99E-09	5.57E-08
Serine protease inhibitor Kazal-type 1	ISK1_HUMAN	−4.212	4.16E-09	5.57E-08
Carboxypeptidase B	CBPB1_HUMAN	−4.187	1.89E-07	7.44E-07

### Comparative Pathway Analysis

**Figure 3:**
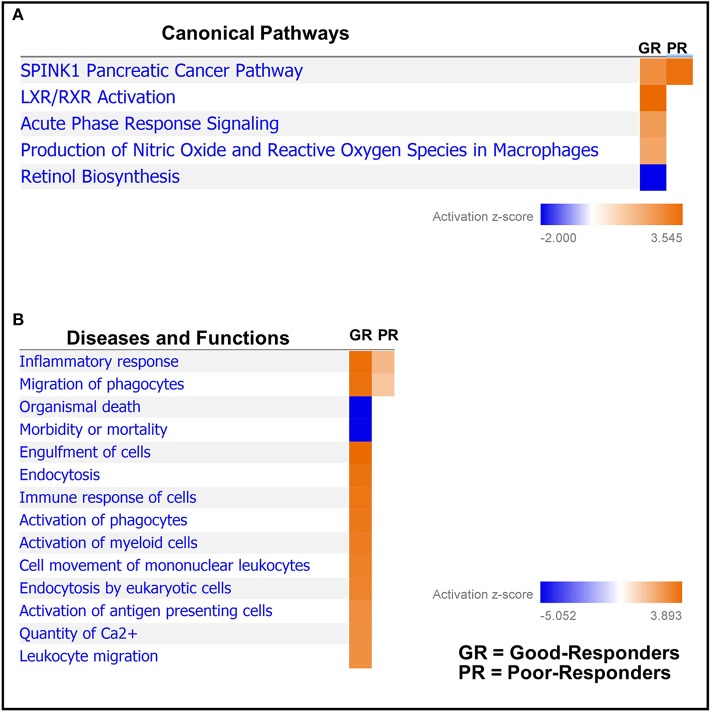
Comparative pathways and associated disease/functions between good- and poor-NAC Responders. Ingenuity pathway analysis was performed to identify **(A)** canonical pathways; and **(B)** associated disease/function affected in the tumor tissue of good- and poor-NAC responding PDAC patients, compared to adjacent normal pancreas.

Next, based on the identified differentially regulated proteins in both good- and poor-NAC responders, pathway analysis was performed using Ingenuity Pathway Analysis. A number of canonical pathways were observed to be differentially regulated in good- and poor-NAC responders (Figure 3A and Supplementary Table 3). Notably, immune response pathways, such as acute phase signaling and macrophage mediated nitric oxide and reactive oxygen species production, were upregulated in good responders but remained unaffected in poor-responders. Similarly, analysis of predicted disease and functions based on differential protein expression using IPA, supported an immunogenic phenotype in good-responders, while poor-responders showed only mild inflammatory response and phagocyte migration (Figure 3B).

### Biomarker Analysis

There were 19 proteins which were markedly (log_2_ > 2) and significantly (*p* < 0.05) over-expressed in tumor from the poor-responders compared to good-responders (Table 3). The ability to these proteins to predict chemo-resistance to NAC was determined by area under the receiver operator characteristic (AUROC) curve. Four biomarkers, namely GRP78, CADM1, PGES2, and RUXF, demonstrated very high predictive performance with AUROC ≥ 0.92.

**Table 3:** Biomarkers to predict poor-NAC response.

**Protein name**	**Uniprot accession**	**Log_**2**_ ratio**	***P*-value**	***q*-value**	**AUROC**
Endoplasmic reticulum chaperone BiP	GRP78_HUMAN	2.139	0.009	0.028	0.954
Cell adhesion molecule 1	CADM1_HUMAN	2.424	0.007	0.028	0.923
Prostaglandin E synthase 2	PGES2_HUMAN	2.359	0.001	0.028	0.923
Small nuclear ribonucleoprotein F	RUXF_HUMAN	2.144	0.009	0.028	0.923
ATP-binding cassette sub-family D member 3	ABCD3_HUMAN	2.060	0.005	0.028	0.892
START domain-containing protein 10	PCTL_HUMAN	2.261	0.006	0.028	0.876
39S ribosomal protein L37	RM37_HUMAN	2.986	0.012	0.030	0.862
Protein enabled homolog	ENAH_HUMAN	2.956	0.003	0.028	0.862
Transmembrane emp24 domain-containing protein 2	TMED2_HUMAN	2.193	0.039	0.061	0.862
Anterior gradient protein 2 homolog	AGR2_HUMAN	2.036	0.027	0.045	0.862
Glutamate decarboxylase 2	DCE2_HUMAN	3.225	0.016	0.034	0.846
Acyl-coenzyme A synthetase ACSM3	ACSM3_HUMAN	2.241	0.013	0.030	0.831
Epiplakin	EPIPL_HUMAN	2.106	0.026	0.045	0.831
SCY1-like protein 2	SCYL2_HUMAN	3.729	0.004	0.028	0.815
Synaptosomal-associated protein 29	SNP29_HUMAN	2.279	0.013	0.030	0.800
Protein JTB	JTB_HUMAN	2.449	0.044	0.062	0.785
MARCKS-related protein	MRP_HUMAN	2.206	0.045	0.062	0.785
60S ribosomal protein L38	RL38_HUMAN	2.061	0.047	0.062	0.785
YTH domain-containing family protein 3	YTHD3_HUMAN	2.175	0.022	0.042	0.738

Notably, four proteins, i.e., TMED2, AGR2, JTB, and CADM1, were predicted as secreted proteins, with SignalP score of 0.908, 0.856, 0.759, and 0.699, respectively.

## Discussion

Neoadjuvant chemotherapy (NAC) is being increasingly given to PDAC patients with borderline/locally advanced disease and is also being evaluated in upfront operable patients. Previous studies have shown that patients who respond to NAC have an overall survival benefit compared to non-responders (7). There are currently no validated biomarkers readily available for predicting NAC response in these patients. This study identified a panel of potential biomarkers which correlate with resistance to NAC in PDAC patients. The top four biomarkers for NAC resistance, namely, GRP78, CADM1, PGES2, and RUXF demonstrated very high predictive ability for chemo-resistance with AUROC > 0.92. Notably, GRP78 has been previously demonstrated to play an important role in mediating chemo-resistance in PDAC (15–17). Moreover, RUXF and PGES2 are known to be involved in chemo-resistance in ovarian and colorectal cancer, respectively (18, 19). On the other hand, CADM1 is shown to be a good prognostic marker in other cancers (20, 21).

Four proteins (i.e., TMED2, AGR2, JTB, and CADM1) among the over-expressed proteins in poor-responders (Table 3) were predicted to be secreted extracellularly. This is important, as detection of these proteins in plasma/serum from the PDAC patients could be used to develop a simple blood-based test for determining NAC response in PDAC patients. Of note, TMED2, AGR2, and JTB are known to be associated with poor prognosis in other cancers (22–24) and thus, could be explored as novel biomarkers for predicting chemo-resistance in PDAC. Future studies, assessing the levels of these biomarkers in serum or plasma from the NAC-treated PDAC patients, will be required to confirm the clinical utility of these biomarkers as an indicator of chemo-resistance in PDAC.

This study also compared tumor tissue with adjacent normal pancreas in both good- and poor- NAC responders. A distinct proteomic profile of over-expressed proteins in tumors was observed in good- and poor-NAC responders compared to the adjacent normal pancreas. Rho guanine nucleotide exchange factor 18 (Uniprot: ARHGI_Human) was the most over-expressed protein in good-responders. This latter protein is known to be up-regulated in response to reactive oxygen species (25), which are known to be increased in tumor tissue treated with chemotherapy (26). Periostin (Uniprot: POSTN) was identified as the highest over-expressed protein in poor-NAC responders. Periostin is an extracellular matrix protein, which is known to play an important role in cancer progression (27). Notably, periostin expression has also been shown to be associated with chemo-resistance in pancreatic and other cancers (28–30). Previous studies have also demonstrated periostin as a poor prognostic biomarker in PDAC and other cancers (31–34). In PDAC, periostin is produced by pancreatic stellate cells and it is shown to establish a microenvironment that is supportive for cancer growth and progression (31, 35). Identification of periostin as a highly over-expressed protein in poor-NAC responders in this study further supports its important role in PDAC chemo-resistance.

The majority of proteins under-expressed in both good- and poor-NAC responders were pancreas specific peptidases or proteases (e.g., CEL3A, CBPB1, CBPA1, CBPA2, etc.). Notably, several previous studies also have shown that pancreatic proteases or peptidases are downregulated in PDAC tumor compared adjacent normal pancreas (33, 36, 37).

Assessment of pathway analysis revealed that the SPINK1 pathway was upregulated in both good- and poor-NAC responders, with the latter having more pronounced involvement of this pathway. SPINK1 is a serine protease inhibitor which has an anti-trypsin activity and is known to play an important role in protecting the normal pancreatic tissue from inadvertent activation of trypsin (38). Moreover, SPINK1 is also shown to play a role in cancer cell survival and progression (39–41). In this study, we observed that levels of SPINK1 (Uniprot: ISK1_Human) were decreased in tumor tissue compared to adjacent normal pancreas. This is consistent with previous studies demonstrating higher levels of SPINK1 in normal pancreatic tissue compared to tumor (42, 43).

The pathway analysis further demonstrated that innate immune response was highly activated in tumors from the good-NAC responders, while only moderate immune activity was observed in poor-NAC responders. It can be postulated that initial response to NAC in good-responders could have resulted in a heightened immune infiltration into these tumors, resulting in an overall increased anti-tumor response. Studies have also shown similar immune-stimulatory effect of chemotherapy in other cancers (44, 45), but this is the first study to observe this effect in PDAC.

The main limitation of this study is a relatively small cohort size. Future multi-institutional studies with a larger group of patients will be required to independently validate the identified proteins and their predictive value. This study utilized tumor specimens obtained at the time of surgical resection after chemotherapy treatment. Future studies will be required to further validate these findings using pre-NAC endoscopic ultrasound (EUS) core biopsies. Notably, EUS core biopsy provides sufficient amount of protein (~1 μg) required for SWATH-MS analysis, which highlights the future clinical utility of these biomarkers in selecting patients for NAC prior to surgery.

## Conclusion

Overall, this exploratory study has demonstrated the successful application of SWATH-MS proteomic analysis to pancreatic tumor and normal pancreas tissue samples, resulting in the identification of novel potential biomarkers which may predict for a chemo-resistant tumor phenotype in PDAC patients treated with NAC. Further research in a larger patient cohort is required to validate these findings.

## Data Availability Statement

The datasets generated for this study can be found in the ProteomeXchange via the PRIDE database (46) (Accession: PXD017051).

## Ethics Statement

The studies involving human participants were reviewed and approved by Royal North Shore Hospital Institutional Ethics Committee, RNSH, St Leonards, Australia and North Shore Private Hospital Institutional Ethics Committee, NSP, St Leonards, Australia. The patients/participants provided their written informed consent to participate in this study.

## Author Contributions

SS, CN, MM, and AM were involved in the design of the study. CN and CK performed the proteomic experiments. AG analyzed the residual tumor viability. SS, SMe, SMa, and AM were involved in the statistical analysis and interpretation of data. MI, NP, SC, DC, JS, and AM enrolled patients. SS and AM wrote the draft manuscript. All authors helped with the manuscript editing. All authors read and approved the final manuscript.

### Conflict of Interest

The authors declare that the research was conducted in the absence of any commercial or financial relationships that could be construed as a potential conflict of interest.

## References

[1] Ilic M Ilic I. Epidemiology of pancreatic cancer. World J Gastroenterol. (2016) 22:9694–705. 10.3748/wjg.v22.i44.9694 27956793 PMC5124974

[2] Rahib L Smith BD Aizenberg R Rosenzweig AB Fleshman JM Matrisian LM. Projecting cancer incidence and deaths to 2030: the unexpected burden of thyroid, liver, and pancreas cancers in the United States. Cancer Res. (2014) 74:2913–21. 10.1158/0008-5472.CAN-14-0155 24840647

[3] Aslan M Shahbazi R Ulubayram K Ozpolat B. Targeted therapies for pancreatic cancer and hurdles ahead. Anticancer Res. (2018) 38:6591–606. 10.21873/anticanres.13026 30504367

[4] Conroy T Hammel P Hebbar M Ben Abdelghani M Wei AC Raoul J-L FOLFIRINOX or gemcitabine as adjuvant therapy for pancreatic cancer. N Engl J Med. (2018) 379:2395–406. 10.1056/NEJMoa1809775 30575490

[5] Roland CL Yang AD Katz MHG Chatterjee D Wang H Lin H. Neoadjuvant therapy is associated with a reduced lymph node ratio in patients with potentially resectable pancreatic cancer. Ann Surg Oncol. (2015) 22:1168–75. 10.1245/s10434-014-4192-6 25352267 PMC5131370

[6] Itchins M Arena J Nahm CB Rabindran J Kim S Gibbs E. Retrospective cohort analysis of neoadjuvant treatment and survival in resectable and borderline resectable pancreatic ductal adenocarcinoma in a high volume referral centre. Eur J Surg Oncol. (2017) 43:1711–7. 10.1016/j.ejso.2017.06.012 28688722

[7] Townend P de Reuver PR Chua TC Mittal A Clark SJ Pavlakis N. Histopathological tumour viability after neoadjuvant chemotherapy influences survival in resected pancreatic cancer: analysis of early outcome data. ANZ J Surg. (2018) 88:E167–72. 10.1111/ans.13897 28318082

[8] Bailey P Chang DK Nones K Johns AL Patch AM Gingras MC. Genomic analyses identify molecular subtypes of pancreatic cancer. Nature. (2016) 531:47–52. 10.1038/nature16965 26909576

[9] Gillet LC Navarro P Tate S Rost H Selevsek N Reiter L. Targeted data extraction of the MS/MS spectra generated by data-independent acquisition: a new concept for consistent and accurate proteome analysis. Mol Cell Proteomics. (2012) 11:O111.016717. 10.1074/mcp.O111.016717 22261725 PMC3433915

[10] Guo T Kouvonen P Koh CC Gillet LC Wolski WE Rost HL. Rapid mass spectrometric conversion of tissue biopsy samples into permanent quantitative digital proteome maps. Nat Med. (2015) 21:407–13. 10.1038/nm.3807 25730263 PMC4390165

[11] Kramer A Green J Pollard J Jr Tugendreich S. Causal analysis approaches in ingenuity pathway analysis. Bioinformatics. (2014) 30:523–30. 10.1093/bioinformatics/btt703 24336805 PMC3928520

[12] Kaspric N Picard B Reichstadt M Tournayre J Bonnet M. ProteINSIDE to easily investigate proteomics data from ruminants: application to mine proteome of adipose and muscle tissues in bovine foetuses. PLoS ONE. (2015) 10:e0128086. 10.1371/journal.pone.0128086 26000831 PMC4441380

[13] Lee SM Katz MH Liu L Sundar M Wang H Varadhachary GR. Validation of a proposed tumor regression grading scheme for pancreatic ductal adenocarcinoma after neoadjuvant therapy as a prognostic indicator for survival. Am J Surg Pathol. (2016) 40:1653–60. 10.1097/PAS.0000000000000738 27631521 PMC5178828

[14] David CC Jacobs DJ. Principal component analysis: a method for determining the essential dynamics of proteins. Methods Mol Biol. (2014) 1084:193–226. 10.1007/978-1-62703-658-0_11 24061923 PMC4676806

[15] Gifford JB Huang W Zeleniak AE Hindoyan A Wu H Donahue TR. Expression of GRP78: master regulator of the unfolded protein response, increases chemoresistance in pancreatic ductal adenocarcinoma. Mol Cancer Ther. (2016) 15:1043–52. 10.1158/1535-7163.MCT-15-0774 26939701

[16] Dauer P Sharma NS Gupta VK Nomura A Dudeja V Saluja A. GRP78-mediated antioxidant response and ABC transporter activity confers chemoresistance to pancreatic cancer cells. Mol Oncol. (2018) 12:1498–512. 10.1002/1878-0261.12322 29738634 PMC6120253

[17] Clarke WR Amundadottir L James MA. CLPTM1L/CRR9 ectodomain interaction with GRP78 at the cell surface signals for survival and chemoresistance upon ER stress in pancreatic adenocarcinoma cells. Int J Cancer. (2019) 144:1367–78. 10.1002/ijc.32012 30468251

[18] Peters D Freund J Ochs RL. Genome-wide transcriptional analysis of carboplatin response in chemosensitive and chemoresistant ovarian cancer cells. Mol Cancer Ther. (2005) 4:1605–16. 10.1158/1535-7163.MCT-04-0311 16227411

[19] Cao B Luo L Feng L Ma S Chen T Ren Y. A network-based predictive gene-expression signature for adjuvant chemotherapy benefit in stage II colorectal cancer. BMC Cancer. (2017) 17:844. 10.1186/s12885-017-3821-4 29237416 PMC5729289

[20] Hartsough EJ Weiss MB Heilman SA Purwin TJ Kugel CH 3rd Rosenbaum SR. CADM1 is a TWIST1-regulated suppressor of invasion and survival. Cell Death Dis. (2019) 10:281. 10.1038/s41419-019-1515-3 30911007 PMC6433918

[21] Ito T Nakamura A Tanaka I Tsuboi Y Morikawa T Nakajima J. CADM1 associates with Hippo pathway core kinases; membranous co-expression of CADM1 and LATS2 in lung tumors predicts good prognosis. Cancer Sci. (2019) 110:2284–95. 10.1111/cas.14040 31069869 PMC6609799

[22] Pan JS Cai JY Xie CX Zhou F Zhang ZP Dong J. Interacting with HBsAg compromises resistance of jumping translocation breakpoint protein to ultraviolet radiation-induced apoptosis in 293FT cells. Cancer Lett. (2009) 285:151–6. 10.1016/j.canlet.2009.05.009 19487072

[23] Lin X Liu J Hu SF Hu X. Increased expression of TMED2 is an unfavorable prognostic factor in patients with breast cancer. Cancer Manag Res. (2019) 11:2203–14. 10.2147/CMAR.S192949 31114314 PMC6497492

[24] Tian SB Tao KX Hu J Liu ZB Ding XL Chu YN. The prognostic value of AGR2 expression in solid tumours: a systematic review and meta-analysis. Sci Rep. (2017) 7:15500. 10.1038/s41598-017-15757-z 29138453 PMC5686151

[25] Yi YW Oh S. Comparative analysis of NRF2-responsive gene expression in AcPC-1 pancreatic cancer cell line. Genes Genomics. (2015). 37:97–109. 10.1007/s13258-014-0253-2 25540678 PMC4269820

[26] Yang H Villani RM Wang H Simpson MJ Roberts MS Tang M. The role of cellular reactive oxygen species in cancer chemotherapy. J Exp Clin Cancer Res. (2018) 37:266. 10.1186/s13046-018-0909-x 30382874 PMC6211502

[27] González-González L Alonso J. Periostin: a matricellular protein with multiple functions in cancer development and progression. Front Oncol. (2018) 8:225. 10.3389/fonc.2018.00225 29946533 PMC6005831

[28] Nakazawa Y Taniyama Y Sanada F Morishita R Nakamori S Morimoto K. Periostin blockade overcomes chemoresistance via restricting the expansion of mesenchymal tumor subpopulations in breast cancer. Sci Rep. (2018) 8:4013. 10.1038/s41598-018-22340-7 29507310 PMC5838092

[29] Liu Y Li F Gao F Xing L Qin P Liang X. Periostin promotes the chemotherapy resistance to gemcitabine in pancreatic cancer. Tumour Biol. (2016) 37:15283–91. 10.1007/s13277-016-5321-6 27696296

[30] Ryner L Guan Y Firestein R Xiao Y Choi Y Rabe C. Upregulation of periostin and reactive stroma is associated with primary chemoresistance and predicts clinical outcomes in epithelial ovarian cancer. Clin Cancer Res. (2015) 21:2941–51. 10.1158/1078-0432.CCR-14-3111 25838397

[31] Erkan M Kleeff J Gorbachevski A Reiser C Mitkus T Esposito I. Periostin creates a tumor-supportive microenvironment in the pancreas by sustaining fibrogenic stellate cell activity. Gastroenterology. (2007) 132:1447–64. 10.1053/j.gastro.2007.01.031 17408641

[32] Dong D Zhang L Jia L Ji W Wang Z Ren L. Identification of serum periostin as a potential diagnostic and prognostic marker for colorectal cancer. Clin Lab. (2018) 64:973–81. 10.7754/Clin.Lab.2018.171225 29945311

[33] Song Y Wang Q Wang D Junqiang L Yang J Li H. Label-free quantitative proteomics unravels carboxypeptidases as the novel biomarker in pancreatic ductal adenocarcinoma. Transl Oncol. (2018) 11:691–9. 10.1016/j.tranon.2018.03.005 29631213 PMC6154863

[34] Tian B Zhang Y Zhang J. Periostin is a new potential prognostic biomarker for glioma. Tumour Biol. (2014) 35:5877–83. 10.1007/s13277-014-1778-3 24719188

[35] Liu Y Li F Gao F Xing L Qin P Liang X. Role of microenvironmental periostin in pancreatic cancer progression. Oncotarget. (2017) 8:89552–65. 10.18632/oncotarget.11533 29163770 PMC5685691

[36] Lu Z Hu L Evers S Chen J Shen Y. Differential expression profiling of human pancreatic adenocarcinoma and healthy pancreatic tissue. Proteomics. (2004) 4:3975–88. 10.1002/pmic.200300863 15526344

[37] Chung JC Oh MJ Choi SH Bae CD. Proteomic analysis to identify biomarker proteins in pancreatic ductal adenocarcinoma. ANZ J Surg. (2008) 78:245–51. 10.1111/j.1445-2197.2008.04429.x 18366394

[38] Mehner C Radisky ES. Bad tumors made worse: SPINK1. Front Cell Dev Biol. (2019) 7:1–5. 10.3389/fcell.2019.00010 30778387 PMC6369215

[39] Ozaki N Ohmuraya M Hirota M Ida S Wang J Takamori H. Serine protease inhibitor Kazal type 1 promotes proliferation of pancreatic cancer cells through the epidermal growth factor receptor. Mol Cancer Res. (2009) 7:1572–81. 10.1158/1541-7786.MCR-08-0567 19737965

[40] Wang C Wang L Su B Lu N Song J Yang X. Serine protease inhibitor Kazal type 1 promotes epithelial-mesenchymal transition through EGFR signaling pathway in prostate cancer. Prostate. (2014) 74:689–701. 10.1002/pros.22787 24619958

[41] Tiwari R Pandey SK Goel S Bhatia V Shukla S Jing X. SPINK1 promotes colorectal cancer progression by downregulating Metallothioneins expression. Oncogenesis. (2015) 4:e162. 10.1038/oncsis.2015.23 26258891 PMC4632074

[42] Marks WH Ohlsson K Polling A. Immunocytochemical distribution of trypsinogen and pancreatic secretory trypsin inhibitor in normal and neoplastic tissues in man. Scand J Gastroenterol. (1984) 19:673–6. 10.1080/00365521.1984.12005792 6382569

[43] Haglund C Huhtala ML Halila H Nordling S Roberts PJ Scheinin TM. Tumour-associated trypsin inhibitor, TATI, in patients with pancreatic cancer, pancreatitis and benign biliary diseases. Br J Cancer. (1986) 54:297–303. 10.1038/bjc.1986.176 3741764 PMC2001515

[44] Sherif A Winerdal M Winqvist O. Immune responses to neoadjuvant chemotherapy in muscle invasive bladder cancer. Bladder Cancer. (2018) 4:1–7. 10.3233/BLC-170123 29430502 PMC5798528

[45] Parra ER Villalobos P Behrens C Jiang M Pataer A Swisher SG. Effect of neoadjuvant chemotherapy on the immune microenvironment in non-small cell lung carcinomas as determined by multiplex immunofluorescence and image analysis approaches. J Immunother Cancer. (2018) 6:48. 10.1186/s40425-018-0368-0 29871672 PMC5989476

[46] Vizcaino JA Cote RG Csordas A Dianes JA Fabregat A Foster JM. The PRoteomics IDEntifications (PRIDE) database and associated tools: status in 2013. Nucleic Acids Res. (2013) 41:D1063–9. 10.1093/nar/gks1262 23203882 PMC3531176

