# Supplementary material for: Identification of Novel Biomarkers in Pancreatic Tumor Tissue to Predict Response to Neoadjuvant Chemotherapy
Source: Front Oncol. 2020 Mar 4;10:237. doi: 10.3389/fonc.2020.00237 (PMC7064619; doi:10.3389/fonc.2020.00237)
Supplement: Supplementary file 1 [file Data_Sheet_1.pdf]

SUPPLEMENTARY DATA

Identification of Novel Biomarkers in Pancreatic Tumor Tissue to Predict Response to Neoadjuvant Chemotherapy

Sumit Sahni,[1,2,3] Christopher Namh,[1,2,3] Chritoph Krisp,[4] Mark Molloy,[1,5] Sarah Maloney,[1,2] Nick Pavlakis,[1,2,6] Stephen Clarke,[1,2,6] David Chan[1,2,6,] Anthony Gill,[1,7] Viive Howell,[1,2] Jaswinder Samra,[1,3,8] and Anubhav Mittal[1,3,8*]

Supplementary Table 1: Differentially Expressed Proteins in Good-NAC Responders

Uniprot Accession	Log Ratio	P value	q value
ARHGI_HUMAN	4.91475328	0.00405	0.011085
PGS1_HUMAN	4.22184924	0.00264	0.009676
PRELP_HUMAN	4.115081402	0.00884	0.016771
CSPG2_HUMAN	4.1090001	0.00275	0.009742
A1AT_HUMAN	3.93542658	0.00694	0.014783
APOA1_HUMAN	3.7349486	0.003	0.009995
HEMO_HUMAN	3.58974688	0.00622	0.013696
CO3A1_HUMAN	3.58773952	0.01233	0.019199
ITIH1_HUMAN	3.53753538	0.00505	0.012446
FBLN1_HUMAN	3.4570102	0.00699	0.014783
C1QA_HUMAN	3.440741816	0.00161	0.00863
GLUC_HUMAN	3.4067368	0.02311	0.030196
C4BPA_HUMAN	3.37152682	0.00034	0.00636
POSTN_HUMAN	3.30036254	0.03526	0.042057
CA050_HUMAN	3.27575544	0.04197	0.047241
IGHG2_HUMAN	3.26443964	0.01841	0.025459
IC1_HUMAN	3.25820406	0.00192	0.008936
A2MG_HUMAN	3.2525976	0.0004	0.006575
SDCB1_HUMAN	3.20974436	0.00102	0.008569
LV403_HUMAN	3.2057812	0.00058	0.007561
CO4A_HUMAN	3.18360302	0.00766	0.015632
APOA_HUMAN	3.18054394	0.01079	0.018275
CERU_HUMAN	3.12643682	0.00169	0.00863
HPT_HUMAN	3.12530236	0.02929	0.03715
VIME_HUMAN	3.1076236	0.02131	0.028126
THY1_HUMAN	3.104766	0.01136	0.018504
THBG_HUMAN	3.0800328	0.01804	0.025088
VTDB_HUMAN	3.048599828	0.00357	0.010421
ANXA2_HUMAN	3.0232188	0.00922	0.016922
CAV1_HUMAN	2.99144922	0.00285	0.009764
BST1_HUMAN	2.9866761	0.00103	0.008569
LV105_HUMAN	2.9554905	0.00153	0.00863
CFAH_HUMAN	2.94883714	0.00518	0.012446
FRIL_HUMAN	2.927183114	0.04797	0.053074
TBCD_HUMAN	2.925749179	0.03686	0.043264
MIME_HUMAN	2.9092328	0.02398	0.03118
LV301_HUMAN	2.88672866	0.00341	0.010195
LPCT4_HUMAN	2.86566457	9.7E-05	0.003142
ASPN_HUMAN	2.82802342	0.008	0.015888
MAP1B_HUMAN	2.81268488	0.00032	0.00635
GOSR1_HUMAN	2.80608774	0.01061	0.018156
RAC2_HUMAN	2.805565436	0.00565	0.012882
U5S1_HUMAN	2.80096276	0.00413	0.011179

NDUB1_HUMAN	2.796244148	0.01122	0.018457
ALBU_HUMAN	2.7749	0.01181	0.018504
HV303_HUMAN	2.7115634	0.0018	0.008849
CO3_HUMAN	2.709734	0.00176	0.008787
IGHM_HUMAN	2.70541372	0.00547	0.012702
EMIL1_HUMAN	2.69621324	0.00499	0.012446
FBN1_HUMAN	2.6947542	0.00748	0.01556
FBLN3_HUMAN	2.69322094	0.01325	0.02026
ANT3_HUMAN	2.6918756	0.00169	0.00863
AMBP_HUMAN	2.69021628	0.00016	0.004606
COEA1_HUMAN	2.67528	0.03345	0.040643
IGHA1_HUMAN	2.65868606	0.0104	0.018032
CO6A3_HUMAN	2.655663	0.03116	0.038215
KNG1_HUMAN	2.64377484	0.00216	0.009498
HV318_HUMAN	2.63811956	0.00863	0.016623
KV205_HUMAN	2.633007454	0.01181	0.018504
THRB_HUMAN	2.6222106	0.00122	0.00863
LV103_HUMAN	2.567662204	0.01089	0.018275
OPLA_HUMAN	2.56661952	0.00905	0.0168
IGKC_HUMAN	2.558065	0.00022	0.005785
1B81_HUMAN	2.540056522	0.00849	0.016563
APOA4_HUMAN	2.5326286	0.00129	0.00863
RAB8A_HUMAN	2.51431848	0.00648	0.014162
TRFE_HUMAN	2.502336	0.00472	0.012152
ITIH2_HUMAN	2.4789125	0.00681	0.014715
DERM_HUMAN	2.46988018	0.01594	0.022895
KV309_HUMAN	2.45327096	0.00145	0.00863
SEC63_HUMAN	2.44748944	0.01573	0.022716
PDLI7_HUMAN	2.44466924	0.00776	0.015632
PLEC_HUMAN	2.4259596	0.00278	0.009742
FA12_HUMAN	2.41826876	0.00527	0.012446
CCAR2_HUMAN	2.41377766	0.01552	0.022543
A1BG_HUMAN	2.40478964	0.00878	0.016771
HSPB6_HUMAN	2.395562112	0.0148	0.021747
FBLN2_HUMAN	2.387571232	0.00384	0.010829
AEBP1_HUMAN	2.38554682	0.00335	0.010195
PPME1_HUMAN	2.38340332	0.01442	0.021302
SFRP4_HUMAN	2.356777086	0.03704	0.043264
FIBB_HUMAN	2.3516214	0.00898	0.0168
APOC3_HUMAN	2.34770898	0.0154	0.022496
AACT_HUMAN	2.3392588	0.00338	0.010195
F13A_HUMAN	2.33526676	0.00125	0.00863
LBP_HUMAN	2.33330382	0.00772	0.015632
GPC6_HUMAN	2.33315076	0.04172	0.047162
A1AG1_HUMAN	2.32044316	0.0192	0.026136
AFAM_HUMAN	2.31240848	0.00257	0.009676

APOA2_HUMAN	2.2918962	0.00169	0.00863
FLNA_HUMAN	2.2891092	0.03105	0.038215
RHG01_HUMAN	2.27476256	0.00466	0.012113
MYOF_HUMAN	2.273105792	0.00134	0.00863
GPX3_HUMAN	2.2697194	0.01347	0.020485
G3P_HUMAN	2.265493	0.03507	0.042016
CO6A1_HUMAN	2.2475104	0.03776	0.043827
ARAP1_HUMAN	2.24737512	0.00281	0.009742
HS12A_HUMAN	2.2361778	0.0029	0.009789
C1QC_HUMAN	2.21162908	0.00525	0.012446
ISCA2_HUMAN	2.20163336	0.01162	0.018504
KLKB1_HUMAN	2.19404032	0.00102	0.008569
PLMN_HUMAN	2.19355358	0.0051	0.012446
SPTB2_HUMAN	2.1891302	0.00739	0.015492
HV309_HUMAN	2.18269092	0.03711	0.043264
PEDF_HUMAN	2.1771712	0.00991	0.017401
NHRF3_HUMAN	2.16224362	8.9E-05	0.003142
LV102_HUMAN	2.1613884	0.00854	0.016563
APOH_HUMAN	2.16054344	0.01058	0.018156
DFNA5_HUMAN	2.1600108	0.00547	0.012702
RAI14_HUMAN	2.14269632	0.01855	0.025514
KCD12_HUMAN	2.142086882	0.00257	0.009676
PCYXL_HUMAN	2.1418768	0.00685	0.014715
COCA1_HUMAN	2.130611384	0.02429	0.031267
ALS_HUMAN	2.129427406	0.00096	0.008569
COG3_HUMAN	2.1282682	0.04579	0.050874
SYPL1_HUMAN	2.12127582	0.0356	0.042266
FBLN5_HUMAN	2.121210492	0.03027	0.037841
NNRE_HUMAN	2.121180186	0.03645	0.04308
VASN_HUMAN	2.11086504	0.01741	0.02447
TGM2_HUMAN	2.09205958	0.00318	0.010195
LUM_HUMAN	2.0801622	0.01392	0.020798
ZCCHV_HUMAN	2.07444156	0.0171	0.024161
ANGT_HUMAN	2.07247948	0.00397	0.010988
RAB8B_HUMAN	2.06119296	0.02771	0.035313
XPP3_HUMAN	2.06004056	0.04143	0.047037
LTBP2_HUMAN	2.055893	0.00056	0.007561
STOM_HUMAN	2.04388172	0.00226	0.009572
IL18_HUMAN	2.029095778	0.01885	0.0258
APOE_HUMAN	2.02704356	0.00144	0.00863
FINC_HUMAN	2.0183666	0.00894	0.0168
SAMP_HUMAN	2.0157782	0.0381	0.044025
LUZP1_HUMAN	2.00929732	0.00146	0.00863
TLN1_HUMAN	2.0050954	0.00103	0.008569
LAMP2_HUMAN	2.00498242	0.03424	0.041219
PACN2_HUMAN	-2.0161112	0.02998	0.037662

RET1_HUMAN	-2.0257645	0.0044	0.011673
TF65_HUMAN	-2.0338438	0.00377	0.010759
HYOU1_HUMAN	-2.0347616	0.00024	0.005785
TMED3_HUMAN	-2.036383172	0.00043	0.006607
RL10_HUMAN	-2.04827014	0.00788	0.01576
HINT1_HUMAN	-2.04904298	0.00924	0.016922
ETFD_HUMAN	-2.0567066	0.00203	0.00925
RT22_HUMAN	-2.06038198	0.00245	0.009676
GABT_HUMAN	-2.06315698	0.03178	0.03879
PRDX4_HUMAN	-2.0802764	0.02947	0.037189
QOR_HUMAN	-2.1150644	0.00527	0.012446
CHMP6_HUMAN	-2.122089008	0.00331	0.010195
RS6_HUMAN	-2.125878	0.02424	0.031267
F162A_HUMAN	-2.13319014	0.00997	0.017401
NDUA7_HUMAN	-2.13707596	0.00341	0.010195
TIM13_HUMAN	-2.14962726	0.00027	0.005818
ACPM_HUMAN	-2.15865478	0.00219	0.009498
K2C5_HUMAN	-2.1594206	0.0043	0.011512
CADM1_HUMAN	-2.164134736	0.01369	0.020687
MACD1_HUMAN	-2.167399865	0.0388	0.044641
RM37_HUMAN	-2.1698618	0.01154	0.018504
CTRL_HUMAN	-2.179233752	0.0013	0.00863
OXA1L_HUMAN	-2.19366796	0.00507	0.012446
S39AE_HUMAN	-2.217285108	0.01111	0.018392
FKBP2_HUMAN	-2.22833872	0.02024	0.027265
GNL1_HUMAN	-2.23398176	0.03109	0.038215
MIC13_HUMAN	-2.235620634	0.00148	0.00863
ATNG_HUMAN	-2.24366544	0.00081	0.008569
ASM3B_HUMAN	-2.26405496	0.04284	0.048006
MT1X_HUMAN	-2.26479684	0.02197	0.028854
HDHD3_HUMAN	-2.273716242	0.039	0.044668
SRBS2_HUMAN	-2.28193984	0.04573	0.050874
CISD1_HUMAN	-2.31627254	0.01307	0.02011
SDF2L_HUMAN	-2.330665244	0.00967	0.017105
GSTA2_HUMAN	-2.33801558	0.04088	0.04662
CLD3_HUMAN	-2.344528852	0.00964	0.017105
AQP8_HUMAN	-2.3460854	0.01381	0.020756
MYCBP_HUMAN	-2.37500684	0.00264	0.009676
JTB_HUMAN	-2.377828	0.00943	0.017024
MGST2_HUMAN	-2.38639915	0.00228	0.009572
PGES2_HUMAN	-2.439430332	0.00444	0.011673
GOT1B_HUMAN	-2.46177292	0.00368	0.010641
TMM65_HUMAN	-2.4655162	0.01975	0.026739
RL12_HUMAN	-2.4733978	0.00103	0.008569
CECR5_HUMAN	-2.4784201	0.00773	0.015632
IDI1_HUMAN	-2.53421952	0.01086	0.018275

TF3C2_HUMAN	-2.53468096	0.03377	0.040841
P5CR1_HUMAN	-2.55310182	0.0026	0.009676
PDIA1_HUMAN	-2.5573208	0.03078	0.038215
SPI2_HUMAN	-2.5784029	0.00078	0.008569
IMPA2_HUMAN	-2.585004388	0.01695	0.024078
K1C18_HUMAN	-2.6016766	0.02108	0.028126
PCTL_HUMAN	-2.619202748	0.01107	0.018392
S61A1_HUMAN	-2.63792614	0.00102	0.008569
CUZD1_HUMAN	-2.64750262	0.00238	0.009676
HM13_HUMAN	-2.68292342	0.00335	0.010195
SSRA_HUMAN	-2.70550472	0.0027	0.009742
PDIA4_HUMAN	-2.7389884	0.02126	0.028126
GAMT_HUMAN	-2.75216318	0.00562	0.012882
PSMD8_HUMAN	-2.7527177	0.0096	0.017105
PPIF_HUMAN	-2.7603326	0.00163	0.00863
REG1B_HUMAN	-2.81018646	0.01157	0.018504
GRP78_HUMAN	-2.8406472	0.00085	0.008569
NDUA5_HUMAN	-2.87386246	0.04941	0.054438
SERC_HUMAN	-2.87560012	0.00189	0.008913
CHM4A_HUMAN	-2.87813328	0.01256	0.019437
KPCD_HUMAN	-2.92876806	0.00591	0.013246
RS27_HUMAN	-2.95340648	0.00387	0.010829
FKB11_HUMAN	-2.96682368	9.3E-05	0.003142
REG3A_HUMAN	-2.975215628	0.01163	0.018504
TMM97_HUMAN	-3.141272372	0.00313	0.010195
GATM_HUMAN	-3.1423898	7.8E-05	0.003142
SPCS3_HUMAN	-3.1782741	8.6E-05	0.003142
CEL3B_HUMAN	-3.21496726	0.00134	0.00863
ENAH_HUMAN	-3.216290544	0.00496	0.012446
ACSM3_HUMAN	-3.23045906	0.00577	0.013046
ERP27_HUMAN	-3.33199764	0.00316	0.010195
LIPP_HUMAN	-3.4528028	0.02112	0.028126
SNP29_HUMAN	-3.47827714	0.0247	0.031641
SDK1_HUMAN	-3.658359114	0.00077	0.008569
REG1A_HUMAN	-3.66386735	0.01776	0.024828
CTRB2_HUMAN	-3.695352474	0.00839	0.016526
ISK1_HUMAN	-3.7337891	0.01407	0.020897
TMED2_HUMAN	-3.7375293	0.00105	0.008569
COL_HUMAN	-3.79752604	0.00219	0.009498
SYCN_HUMAN	-3.81367952	0.00156	0.00863
AMY2B_HUMAN	-3.83859796	0.00057	0.007561
PA21B_HUMAN	-4.03999606	0.00596	0.013255
CEL2A_HUMAN	-4.0965423	0.00184	0.008877
TRY3_HUMAN	-4.14011204	0.01622	0.023172
MT1G_HUMAN	-4.18080764	0.00931	0.016929
CTRC_HUMAN	-4.248910828	0.00348	0.010279

CBPA2_HUMAN	-4.395077726	0.00125	0.00863
CBPA1_HUMAN	-4.7481592	0.01179	0.018504
PDIA2_HUMAN	-4.7574576	0.00252	0.009676
CEL_HUMAN	-4.7752732	0.0025	0.009676
LIPR2_HUMAN	-4.8471826	0.00037	0.00636
TRY1_HUMAN	-5.34629467	0.00126	0.00863
CBPB1_HUMAN	-5.4275338	5.8E-05	0.003142
CEL3A_HUMAN	-6.24516988	4.5E-05	0.003142
AMYP_HUMAN	-6.332573814	4.2E-05	0.003142

Supplementary Table 2: Differentially Expressed Proteins in Poor-NAC Responders

Uniprot Accession	Log Ratio	P value	q value
POSTN_HUMAN	2.788523408	6.9392E-06	1.45289E-05
FLNA_HUMAN	2.700252	3.43608E-05	5.11594E-05
RAC2_HUMAN	2.613402405	2.80093E-07	9.3831E-07
PSB10_HUMAN	2.555261334	3.30695E-05	5.03558E-05
COCA1_HUMAN	2.481921647	2.64083E-07	9.31241E-07
CSPG2_HUMAN	2.435636615	6.8026E-07	1.98163E-06
CO5A2_HUMAN	2.388444954	0.000370535	0.000459737
PGS1_HUMAN	2.373071777	0.000430913	0.000524931
APOA1_HUMAN	2.353701592	0.00075606	0.000830427
SDCB1_HUMAN	2.344668769	6.97043E-05	9.15723E-05
TSP2_HUMAN	2.23919759	5.38792E-05	7.36716E-05
FINC_HUMAN	2.214157308	1.12944E-07	5.82096E-07
PRTN3_HUMAN	2.210374047	0.017665048	0.0179327
PDLI7_HUMAN	2.207382623	4.72283E-05	6.59228E-05
CAPG_HUMAN	2.169465731	1.71085E-05	2.93915E-05
PRELP_HUMAN	2.130257462	0.000492005	0.000558718
FBLN1_HUMAN	2.087093692	0.000483147	0.000558118
ITIH1_HUMAN	2.084038115	2.50159E-05	3.99063E-05
CO3A1_HUMAN	2.072285369	0.001117125	0.001207215
SRP14_HUMAN	2.058143827	0.000442222	0.000528941
IGHG2_HUMAN	2.055155918	0.025951682	0.025951682
RAB8A_HUMAN	2.028541172	0.001603656	0.001705476
MFS10_HUMAN	2.005954646	0.000449994	0.000528941
FBX2_HUMAN	-2.00353906	9.37744E-08	5.23574E-07
HYEP_HUMAN	-2.004542538	2.83935E-06	6.55988E-06
TRY2_HUMAN	-2.082392041	1.65463E-05	2.91737E-05
CUZD1_HUMAN	-2.098173497	9.13019E-05	0.000117639
MMP7_HUMAN	-2.174187608	0.003048863	0.003191779
FABP4_HUMAN	-2.276174727	0.000504073	0.000562881
REG3A_HUMAN	-2.308212382	0.009328169	0.00961519
ECHD3_HUMAN	-2.356878799	1.85759E-08	1.77798E-07
SERC_HUMAN	-2.369977062	1.44518E-07	6.45512E-07
GABT_HUMAN	-2.378204185	2.71672E-06	6.50072E-06
SYCN_HUMAN	-2.381264212	3.04808E-05	4.74934E-05
GATM_HUMAN	-2.394423923	7.71754E-06	1.56689E-05
IMPA2_HUMAN	-2.403002661	4.11404E-09	5.57049E-08
SDK1_HUMAN	-2.420333184	2.03565E-05	3.32655E-05
HBB_HUMAN	-2.451814146	0.000358949	0.000453766
CTRL_HUMAN	-2.562712085	1.47824E-06	3.96168E-06
KLK1_HUMAN	-2.765988944	1.06862E-05	2.04564E-05
MT1X_HUMAN	-2.840429687	1.9929E-05	3.32655E-05
COL_HUMAN	-2.853346231	1.33964E-07	6.41112E-07

GP2_HUMAN	-2.869329946	3.97062E-05	5.78329E-05
GAMT_HUMAN	-3.021948646	4.44119E-08	2.9756E-07
PA21B_HUMAN	-3.057870085	4.0031E-06	8.71617E-06
CEL3B_HUMAN	-3.150653296	2.17803E-06	5.40474E-06
TMM97_HUMAN	-3.232321	3.67059E-07	1.12241E-06
ERP27_HUMAN	-3.2837919	7.57651E-08	4.61478E-07
MT1G_HUMAN	-3.509090438	1.1522E-05	2.08641E-05
REG1A_HUMAN	-3.552767124	6.63617E-05	8.89247E-05
CEL2A_HUMAN	-3.5784439	1.7917E-06	4.61706E-06
LIPR2_HUMAN	-3.593077068	1.11032E-05	2.06644E-05
LIPP_HUMAN	-3.597067385	2.53419E-07	9.31241E-07
CEL_HUMAN	-3.744881554	8.18692E-07	2.28552E-06
AMY2B_HUMAN	-3.787797882	8.93845E-06	1.7614E-05
GSTA2_HUMAN	-3.896438724	3.68552E-07	1.12241E-06
CBPA2_HUMAN	-3.934637085	1.57734E-07	6.60513E-07
CBPB1_HUMAN	-4.186655654	1.88758E-07	7.43927E-07
ISK1_HUMAN	-4.212143322	4.15708E-09	5.57049E-08
CTRC_HUMAN	-4.389588408	2.98828E-09	5.57049E-08
CBPA1_HUMAN	-4.462589692	4.03285E-06	8.71617E-06
TRY1_HUMAN	-4.470508631	2.17627E-09	5.57049E-08
PDIA2_HUMAN	-4.858943438	2.19434E-08	1.83776E-07
CEL3A_HUMAN	-4.933007792	2.3497E-09	5.57049E-08
AMYP_HUMAN	-4.98768344	3.62712E-08	2.70019E-07
CTRB2_HUMAN	-5.067355577	4.55609E-05	6.49485E-05
TRY3_HUMAN	-5.124411762	1.41273E-08	1.57755E-07

Supplementary Table 3: List of Proteins Involved in Identified Canonical Pathways

Canonical Pathway	Proteins Involved	
	Good Responders	Poor Responders
**SPINK1 Pancreatic Cancer Pathway**	↓CELA2A, ↓CELA3A, ↓CELA3B, ↓CLPS, ↓CPA1, ↓CPA2, ↓CPB1, ↓CTRB2, ↓CTRC, ↓CTRL, ↓PRSS1, ↓PRSS3, ↓SPINK1, ↑KLKB1	↓CELA2A, ↓CELA3A, ↓CELA3B, ↓CLPS, ↓CPA1, ↓CPA2, ↓CPB1, ↓CTRB2, ↓CTRC, ↓CTRL, ↓KLK1, ↓PRSS1, ↓PRSS2, ↓PRSS3, ↓SPINK1
**LXR/RXR Activation**	↑APOC3, ↑APOE, ↑APOH, ↑C3, ↑C4A/C4B, ↑GC, ↑HPX, ↑IL18, ↑KNG1, ↑LBP, ↑LPA, ↑ORM1, ↑SERPINA1, ↑SERPINF1, ↑TF, ↓RELA	--
**Acute Phase Response Signaling**	↑A2M, ↑AGT, ↑ALB, ↑AMBP, ↑APCS, ↑APOA1, ↑APOA2, ↑APOH, ↑C3, ↑C4A/C4B, ↑C4BPA, ↑CP, ↑F2, ↑FGB, ↑FN1, ↑FTL, ↑HP, ↑HPX, ↑IL18, ↑ITIH2, ↑KLKB1, ↑LBP, ↑ORM1, ↑PLG, ↑SERPINA1, ↑SERPINA3, ↑SERPINF1, ↑SERPING1, ↑TF, ↓RBP1, ↓RELA	--
**Production of Nitric Oxide and Reactive Oxygen Species in Macrophages**	↑ALB, ↑APOA1, ↑APOA2, ↑APOA4, ↑APOC3, ↑APOE, ↑LPA, ↑ORM1, ↑RAC2, ↑SERPINA1, ↓PRKCD, ↓RELA	--
**Retinol Biosynthesis**	↓CEL, ↓PNLIP, ↓PNLIPRP2, ↓RBP1	↓CEL, ↓PNLIP, ↓PNLIPRP2
